# Utilization of a novel digital measurement tool for quantitative assessment of upper extremity motor dexterity: a controlled pilot study

**DOI:** 10.1186/1743-0003-11-121

**Published:** 2014-08-13

**Authors:** Ruth Getachew, Sunghoon I Lee, Jon A Kimball, Andrew Y Yew, Derek S Lu, Charles H Li, Jordan H Garst, Nima Ghalehsari, Brian H Paak, Mehrdad Razaghy, Marie Espinal, Arsha Ostowari, Amir A Ghavamrezaii, Sahar Pourtaheri, Irene Wu, Majid Sarrafzadeh, Daniel C Lu

**Affiliations:** Neuroplasticity and Repair Laboratory, UCLA, Los Angeles, USA; Neuromotor Recovery and Rehabilitation Center, UCLA, Los Angeles, USA; Computer Science Department, UCLA, Los Angeles, USA; Department of Anesthesia, UCLA, Los Angeles, USA; Department of Neurosurgery, UCLA, Los Angeles, USA; Department of Orthopaedic Surgery, UCLA, Los Angeles, USA

**Keywords:** Cervical spondylosis, Handgrip, Motor deficit, Oswestry disability index

## Abstract

**Background:**

The current methods of assessing motor function rely primarily on the clinician’s judgment of the patient’s physical examination and the patient’s self-administered surveys. Recently, computerized handgrip tools have been designed as an objective method to quantify upper-extremity motor function. This pilot study explores the use of the MediSens handgrip as a potential clinical tool for objectively assessing the motor function of the hand.

**Methods:**

Eleven patients with cervical spondylotic myelopathy (CSM) were followed for three months. Eighteen age-matched healthy participants were followed for two months. The neuromotor function and the patient-perceived motor function of these patients were assessed with the MediSens device and the Oswestry Disability Index respectively. The MediSens device utilized a target tracking test to investigate the neuromotor capacity of the participants. The mean absolute error (MAE) between the target curve and the curve tracing achieved by the participants was used as the assessment metric. The patients’ adjusted MediSens MAE scores were then compared to the controls. The CSM patients were further classified as either “functional” or “nonfunctional” in order to validate the system’s responsiveness. Finally, the correlation between the MediSens MAE score and the ODI score was investigated.

**Results:**

The control participants had lower MediSens MAE scores of 8.09*%*±1.60*%*, while the cervical spinal disorder patients had greater MediSens MAE scores of 11.24*%*±6.29*%*. Following surgery, the functional CSM patients had an average MediSens MAE score of 7.13*%*±1.60*%*, while the nonfunctional CSM patients had an average score of 12.41*%*±6.32%. The MediSens MAE and the ODI scores showed a statistically significant correlation (*r*=-0.341, *p*<1.14×10^-5^). A Bland-Altman plot was then used to validate the agreement between the two scores. Furthermore, the percentage improvement of the the two scores after receiving the surgical intervention showed a significant correlation (*r*=-0.723, *p*<0.04).

**Conclusions:**

The MediSens handgrip device is capable of identifying patients with impaired motor function of the hand. The MediSens handgrip scores correlate with the ODI scores and may serve as an objective alternative for assessing motor function of the hand.

**Electronic supplementary material:**

The online version of this article (doi:10.1186/1743-0003-11-121) contains supplementary material, which is available to authorized users.

## Introduction

Cervical spondylotic myelopathy (CSM) is an age-related form of spinal degeneration. It is the most common cause of spinal pathology in patients over the age of 50[1–3]. Most individuals with CSM have radiographic evidence of cervical spine degenerative changes by the age of 30. The incidence of CSM increases until the age of 60[4, 5]. As the elderly population grows, CSM is expected to become an increasingly common problem facing patients and the healthcare system[6, 7]. CSM is known to impair the neuromotor function of the hand, thus impairing the performance of many daily activities. CSM can cause the impairment of both the upper and lower extremities. The symptoms commonly include motor weakness, pain, and numbness. Consequently, CSM can have a serious impact on the quality of life of the elderly population. For this reason, timely diagnosis is critical[5–7].

The current methods for the clinical evaluation for neuromotor deficits rely on scoring observable changes in neuromuscular function[2, 8]. These physical exam maneuvers include the Barre test, the Hoffmann test, the pronator drift test, the hand withdrawal reflex, and the deep tendon reflexes[9, 10]. The American Spinal Injury Association (ASIA) motor score is another method of determining the neuromotor function of patients, as it assesses the strength of selected muscles against resistance. Unfortunately, the aforementioned physical exam maneuvers are limited by physician bias and by the subjective nature of their scoring systems[2, 11]. Furthermore, the negative predictive value (the number of patients who have a negative test result that is consistent with a negative history of a given diagnosis) of these tests is low, making it difficult to rule out neuromotor deficits based solely on these methods[11]. Radiographic imaging combined with clinical exam findings have become the standard of care in diagnosing spinal cord pathology. However, radiographic imaging is expensive and often does not correlate with functional impairment. Other scoring systems based on activities of daily living have become widely used in assessing CSM patients. These scoring systems include the Japanese-Orthopaedic-Association-Score (JOA-Score)[12] and the Oswestry Disability Index (ODI)[13]. However, these methods are not sensitive to small changes in neuromotor function[14]. Some of these methods, furthermore, are known to be affected by response shift, which represents a change in the standard of perceived health-related quality of life after receiving medical treatment[15].

Systems that objectively measure neuromuscular function, such as the MediSens system, have gained popularity for assessing neurodegenerative disorders[11, 16]. These systems require the patient to perform “tracking maneuvers” by varying their handgrip strength to follow a target curve on a computer. The goal of the test is to minimize the error between the target and the patient’s tracking response. Handgrip motor function tests were performed in stroke and Parkinson’s disease (PD) patients as a method of assessing their function before and after physical rehabilitation[17–19]. Recent studies have identified the sinusoidal curve tracking test as the most useful tracking performance test for patients with neurodegenerative disorders[20]. Studies specific to the sinusoidal curve have also demonstrated its ability to detect performance deficits in the unaffected side of hemi-paraplegic patients when compared to their healthy counterparts[21, 22]. To the best of our knowledge, handgrip tracking devices have not been used to compare the preoperative and the postoperative handgrip performance in patients with CSM.

In this paper, we introduce the MediSens handgrip device as an objective test to assess the neuromotor function. We validate its clinical usefulness by examining the ability of the device in quantifying the neuromotor levels of CSM patients and healthy participants, by examining the capacity of the MediSens handgrip to track changes in hand performance of CSM patients after receiving cervical decompression surgery (i.e., system’s responsiveness), and by investigating its correlation to the patients’ perceived motor deficits in performing daily activities (i.e., the ODI).

## Materials and methods

### Participants

Eleven adult research participants who were scheduled for elective cervical decompression surgery by DCL at the UCLA Spine Center were recruited for this study. The inclusion criteria included age (19-89 years old) and no prior history of cervical pathology and/or neuromuscular impairments. The mean age of the eleven study participants was 58 (39-86). The mean age of the eighteen control subjects was 63 (45-85). Informed consent was obtained from all participants. A medical chart review was performed and all eleven patients were noted to have documented symptoms of myelopathy manifested by upper extremity paresthesia and radiculopathy. A review of preoperative imaging was performed which demonstrated radiographic evidence of cervical spondylotic disease, including spinal canal stenosis, osteophytic disease, facet arthropathy, and ligamentous hypertrophy. This study was reviewed and approved by the UCLA Institutional Review Board.

### MediSens handgrip device

**Figure 1:**
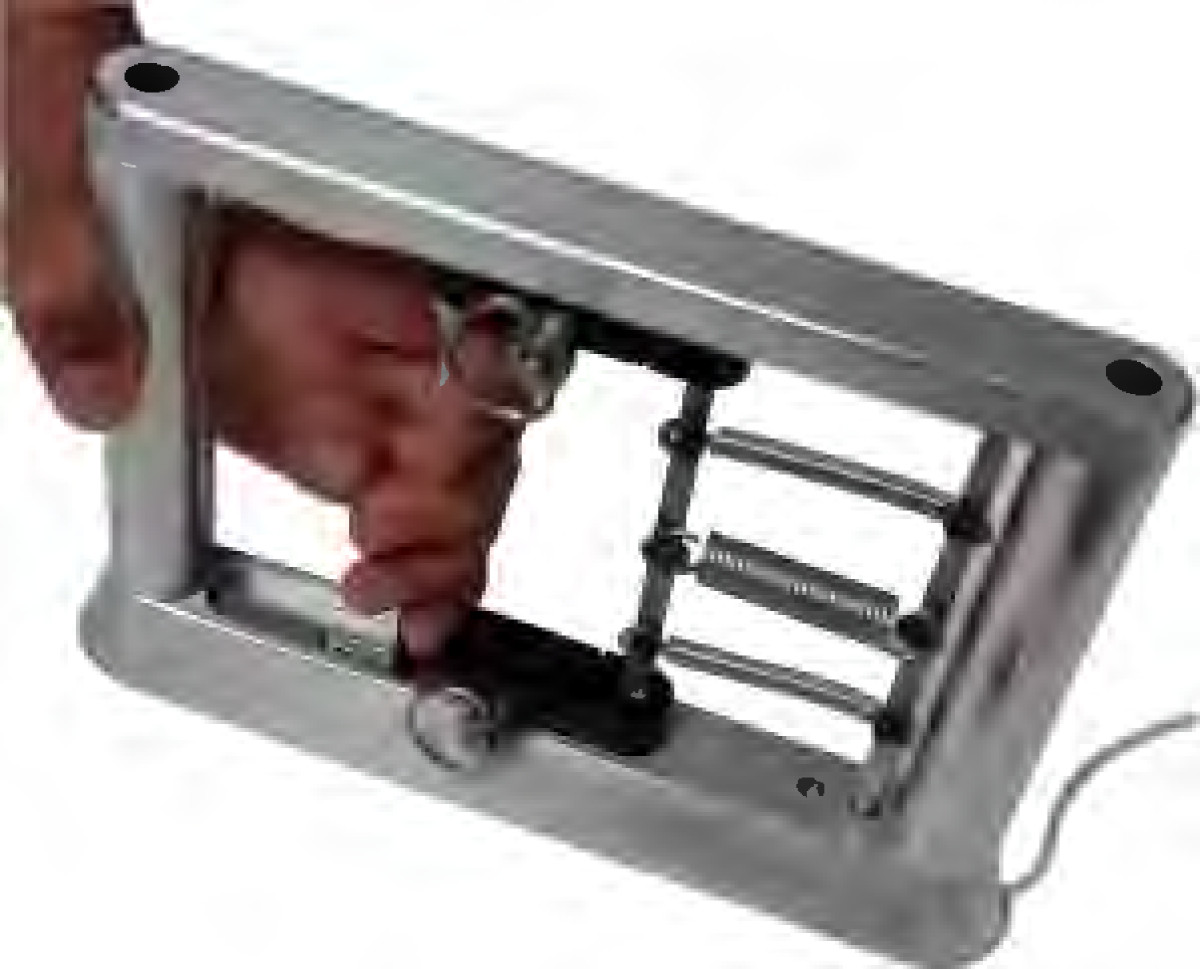
**The MediSens handgrip device used in this work, which has also been introduced in**[23]**.**

Each participant was assessed for neuromotor function of the clinically affected hand with the MediSens system. This handgrip device delivers sensory information to a computer which enables visualization of the patient’s handgrip performance and collects sensory input data.The MediSens handgrip device in Figure1 has three components: the handle, the springs, and a displacement sensor. The main body is composed of three rods and a bottom compartment. The handle is connected to the main body by three springs. These springs create resistance to grasping motions. Five different springs with the following spring constants were used to calibrate the MediSens device with the patient’s hand grip strength: S1 (0.38 lbs/inch), S2 (0.88 lbs/inch), S3 (1.94 lbs/inch), S4 (5.10 lbs/inch), and S5 (10.70 lbs/inch).

Test administrators were not blind to the treatment group. As an attempt to maintain continuity with the patient and to reduce performance variability due to changes in test administrators, each test administrator was responsible for testing the same study participants. Testing methods were the same for both the control and the treatment groups.

### Testing procedure

**Figure 2:**
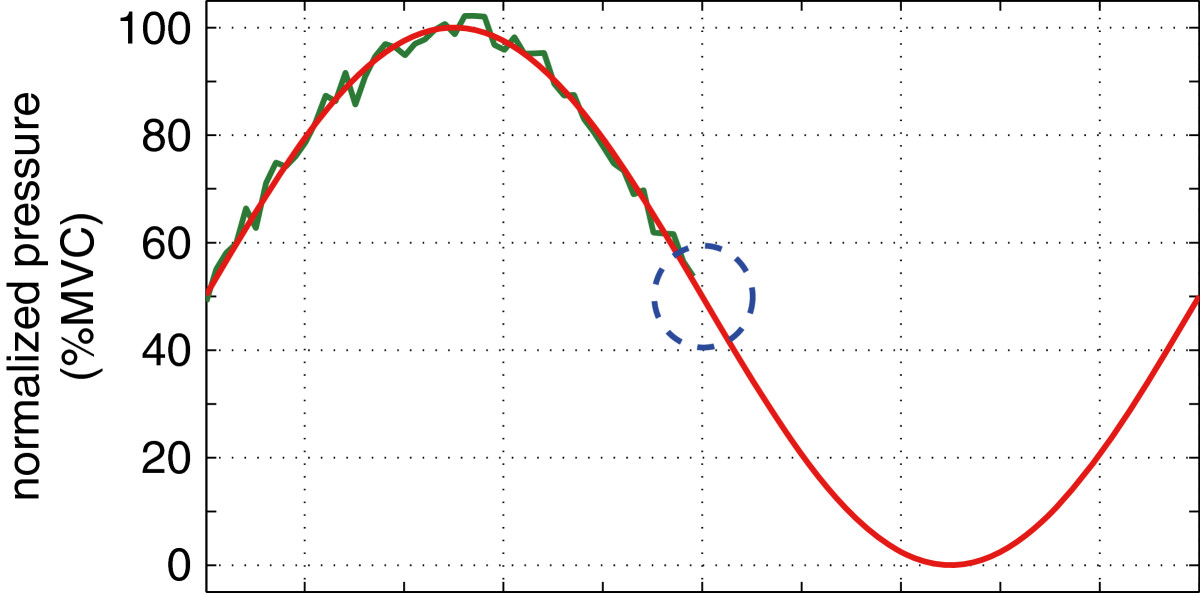
**Example illustration of the target tracking test used in the MediSens system**[23]**.**

In order to calibrate the MediSens handgrip device with the subjects’ grip strength, a set of three springs were chosen to provide resistance equal to 70-80% of the subject’s maximum voluntary contraction (MVC). The tracking tests, as shown in Figure2, were normalized to the individual’s MVC such that the maximum magnitude of the tracking target is equal to the measured MVC.The sinusoidal target moved towards the left of the screen at a constant speed. The dotted circle in Figure2 moved in the y-axis according to the grip force exerted by the subject. The software also displayed the history of the subject’s performance. The objective of the test was to minimize the errors between the target waveform and the dotted circle. At every clinical visit, participants performed three 45-second tracking tests. The three MediSens mean absolute error (MAE) scores from a given test date were averaged to represent the overall performance for that clinical visit.

Longitudinal tracking of the patients’ hand motor function was performed an hour prior to surgery and again at 24 hours, 48 hours, 2 weeks, and 3 months postoperatively. However, only the preoperative and 3-month postoperative data points were used in this study, due to the results of previous studies that did not demonstrate significant improvement in neuromotor function until 3-4 months postoperatively[15]. During the postoperative period, patients were given a cervical collar and were prescribed hand physical therapy as needed. Testing for controls was conducted at 0 weeks, 2-3 weeks and 4-6 weeks.

### Analysis

The MediSens MAE score was used to quantify motor function. It was calculated by averaging the distance between the target curve and the curve tracing achieved by a patient. The sinusoidal test was normalized to the MVC of the subject. Thus, the unit of the MediSens MAE score is % MVC.

In order to track the change in motor function secondary to surgical intervention, a percentage improvement (% Improvement) of the MediSens MAE score was computed using (*MAE*_*post*_-*MAE*_*pre*_)/*MAE*_*pre*_.

### ODI surveys

The ODI survey consists of ten questions regarding the patient’s ability to perform the activities of daily living[24, 25]. The answer choices describe a progressively increasing level of neuromotor impairment that correlates with a higher numeric score. The scores on the 10 questions were averaged to create a numeric value that indicates the patient’s perceived level of motor function.

ODI scores were collected from CSM patients preoperatively and at three months postoperatively. Patients with a three month postoperative ODI score ≤ 0.6 were categorized as “functional”, while patients with three month postoperative ODI score > 0.6 were categorized as “nonfunctional”[13]. This threshold-based categorization is an approximated representation of a patient group (i.e., ≤ 0.6) that is relatively functional in performing daily activities compared to the rest of the patients (i.e., > 0.6)[13].

Written informed consent was obtained from all patients for publication of this study. A copy of the written consents are available for review by the Editor-in-Chief of this journal.

The study was performed in accordance with the Declaration of Helsinki, the protocol was approved by the Human Research Protection Program at UCLA and all patients or their carers gave informed consent. This research was approved by the University of California, Los Angeles, Institutional Review Board (IRB #: 12-000009-CR-00002).

## Results

Table1 describes the demographics of the current study. 18 control participants and 11 surgical patients were enrolled, with mean ages of 63 and 58 years respectively.

**Table 1:** **Demographic information about the participants**

	Control (n = 18)	CSM Patients (n = 11)
Age range	45-85	39-86
Mean age	63 ± 3.38	58 ± 3.20
Mean weight	149 ± 7.93	166 ± 9.84
Mean gender	33%	63%
Cervical neck injury	-	1 (9%)
Cervical neck degenerative	-	10 (91%)
symptoms		

### The MediSens MAE scores of CSM patients and control subjects

Each participant performed the tracking test three times per clinical visit. The average MediSens MAE score of the three tests was used to quantify the hand neuromuscular function for that test date. Table2 summarizes the average scores of the control participants at 0 weeks and at the two-month mark. It also summarizes the average MediSens MAE scores of the CSM patients preoperatively and at three months postoperatively. The table further reports the average MediSens MAE scores of the postoperative CSM patients divided into functional or nonfunctional groups as described in Section ‘ODI surveys’.

**Table 2:** **A summary of the MediSens MAE scores of control subjects and CSM patients**

		Mean MediSens MAE ± SD
Control	First Test	8.09±1.60
	Last Test	6.21±1.51
CSM Patients	Preoperative test	11.24±6.29
	3 month postoperative	8.71±4.17
	test (aggregate)	
	3 month postoperative	7.13±1.60
	test (functional group)	
	3 month postoperative	12.41±6.32
	test (nonfunctional group)	

The average MediSens MAE score for the control participants was 8.09 ± 1.60 at week 0 and was 6.21 ± 1.51 at the two-month follow-up. For the CSM patients, the preoperative and the three months postoperative tests revealed average MediSens MAE scores of 11.24 ± 6.29 and 8.71 ± 4.17 respectively. The CSM patients were categorized into functional and nonfunctional groups based on the assessment method of Fairbanks et al.[13]. The functional group demonstrated a postoperative MediSens MAE score of 7.13 ± 1.60, while the nonfunctional group showed a score of 12.41 ± 6.32.

The percentage change between the first and the last test sessions was quantified to further demonstrate the change in handgrip performance. Control participants improved 17% ± 15% after two months. This demonstrates a general pattern of motor learning. In contrast, CSM patients improved 21% ± 27% during the three months following surgery. This shows that the motor function of CSM patients improved more than the expected motor learning. This finding suggests a benefit to surgical intervention. Interestingly, in Table3, the functional group improved by 30% ± 18%, while nonfunctional group members declined in performance by 11% ± 25%. Possible causes for the discrepancy in performance between the functional and nonfunctional groups are explored in the discussion.

**Table 3:** **Percentage change in the MediSens MAE score between initial and final time points**

	% Improvement	T-test vs.
	MediSens MAE	control
Control	17*%*±15*%*	-
CSM Patients - Aggregated	21*%*±27*%*	*p*<0.88
CSM Patients - Functional	30*%*±18*%*	*p*<0.09
CSM Patients - Nonfunctional	-11*%*±25*%*	*p*<0.16

### Correlation to existing methods

**Figure 3:**
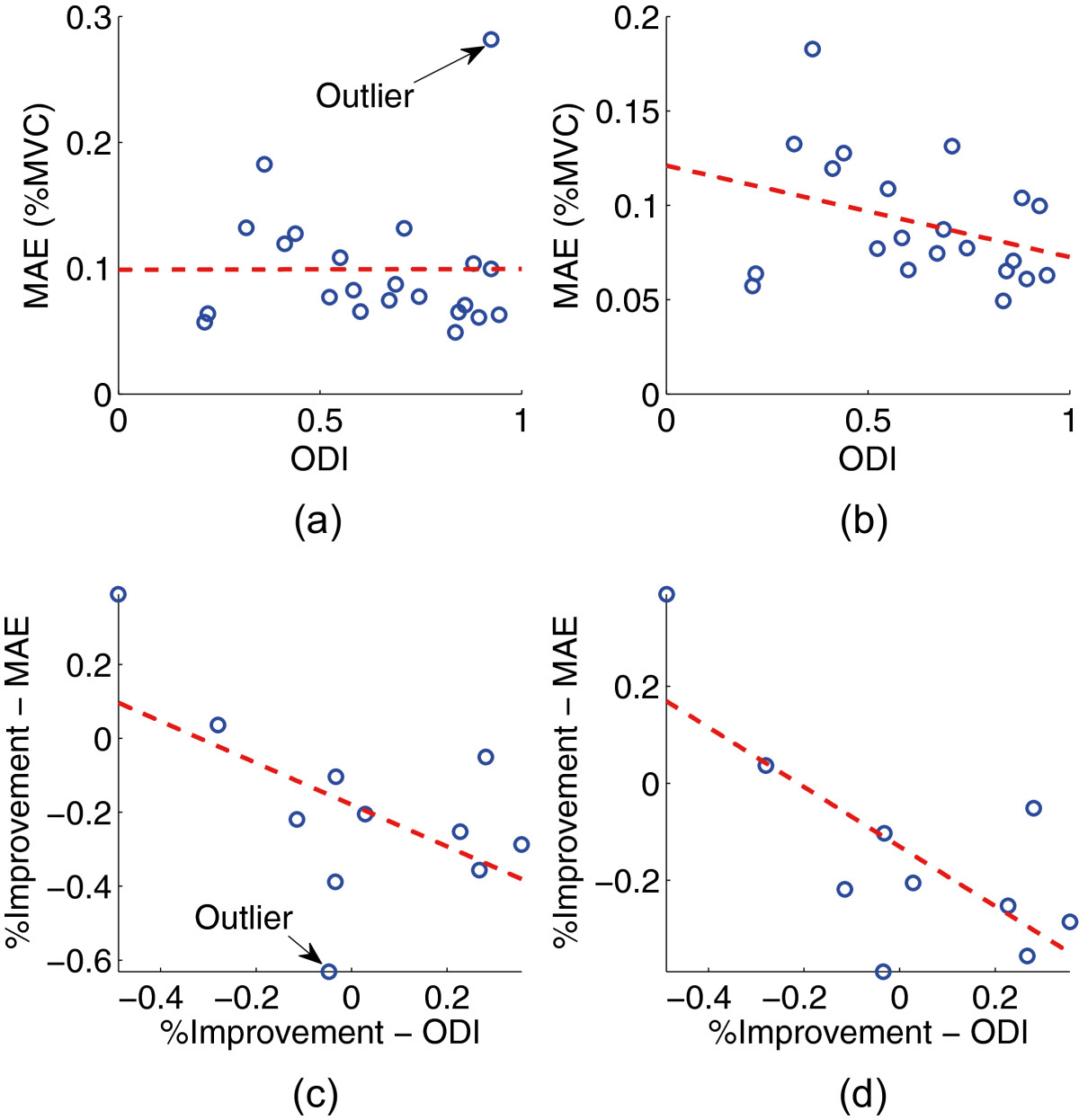
**Various correlation results between the ODI and the MediSens MAE scores.** **(a)** Correlation between the ODI and the MediSens MAE scores, **(b)** Correlation between the ODI and the MediSens MAE scores with the outlier removed, **(c)** Correlation between % Improvement of the ODI and the MediSens MAE scores, **(d)** Correlation between % Improvement of the ODI and the MediSens MAE scores with the outlier removed.

**Figure 4:**
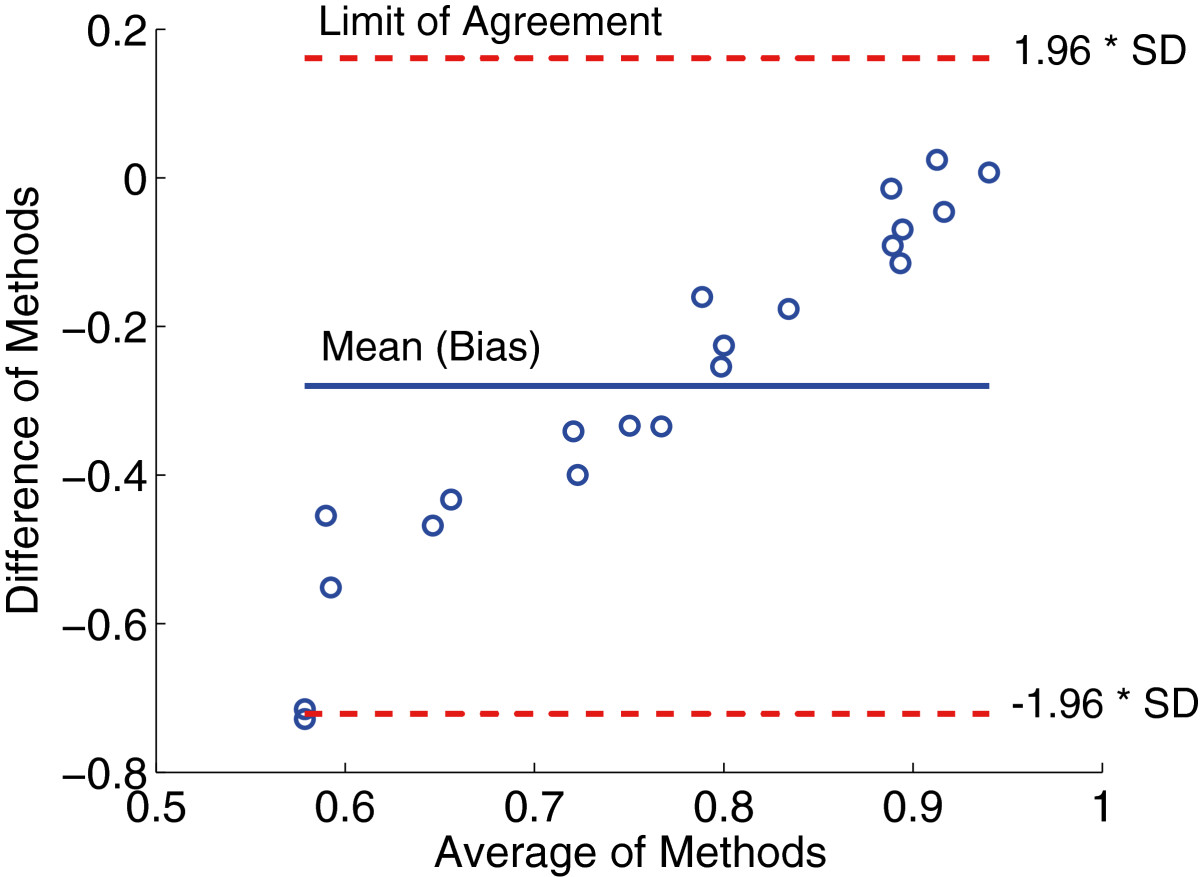
**Bland-Altman plot between the ODI and the MediSens MAE scores.** The x-axis represents the average value of the two scores and the y-axis represents the difference of the two scores.

The ODI surveys were administered at each MediSens handgrip testing and compared to the MediSens MAE scores. This tested our hypothesis that there exists a correlation between ODI scores and handgrip performance. It also tested our hypothesis that a postoperative improvement in ODI correlates with an objective improvement in handgrip neuromuscular function as demonstrated by the MediSens device. The Pearson product-moment correlation coefficient (or Pearson’s *r*) was used to quantify the level of correlation[26–28]. Figure3(a) demonstrates the pre and postoperative correlation between the MediSens MAE and the ODI scores of the surgical patients (*r*=0.0025, *p*<0.00806). The Cook’s distance (*d*_*i*_) of each point was used to identify any possible outliers, which was calculated using the following equation:

$$d_{i}=\frac{\overset{n}{\underset{j=1}{\sum }}{\left({\hat{\text{MAE}}}_{j}-{\hat{\text{MAE}}}_{j-i}\right)}^{2}}{(k+1)s^{2}},i=1,\ldots ,\mathrm{n.}$$

where *n* is the total number of data points,${\hat{\text{MAE}}}_{j}$ represents the regression estimate of the MediSens MAE score at point *j*, and${\hat{\text{MAE}}}_{j-i}$ represents the regression estimate of the MediSens MAE score at point *j* when the data point *i* is removed. Furthermore, *s* represents the estimated root mean square error. A data point is labeled as an outlier if$d_{i}\geq \frac{4}{n-(k+1)}$ where *k* is the number of fitted parameters in our linear model (i.e., *k*=1). As a consequence, one data point, which is labeled in Figure3(a), has been verified as an outlier. Figure3(b) represents the correlation between the MediSens MAE and ODI scores when the outlier is removed. Both the *r* and *p*-value have been significantly improved (*r*=-0.341, *p*<1.14×10^-5^). A Bland-Altman plot was constructed in order to verify the agreement between the MediSens MAE and the ODI scores as shown in Figure4. The mean difference was -0.26 (95% C.I. of -0.36 and -0.15), the upper limit of agreement was 0.22, and the lower limit of agreement was -0.73. Percentage improvement in the ODI scores also displayed a strong correlation to the percentage improvement in the MediSens MAE scores as shown in Figure3(c) (*r*=-0.553, *p*<0.0300). The predefined outlier was removed and the result is illustrated in Figure3(d). The correlation coefficient was significantly improved to *r*=-0.723, *p*<0.0402. These results are also summarized in Table4.

**Table 4:** **Correlation between the ODI and the MediSens MAE scores**

	*r*	*p*-value
MediSens MAE vs. ODI (with outlier)	0.00250	0.008
MediSens MAE vs. ODI (without outlier)	-0.341	1.14×10^-5^
% Improvement MediSens MAE vs. ODI	-0.553	0.03
(with outlier)		
% Improvement MediSens MAE vs. ODI	-0.723	0.04
(without outlier)		

## Discussion

### Method of quantifying MediSens handgrip performance

The MediSens MAE scores demonstrated a significant difference between the control participants and the preoperative CSM patients. Table2 shows that the control participants had an average MediSens MAE score of 8.09 ± 1.60 at the initial attempt, whereas the preoperative CSM patients had an average score 11.24 ± 6.29. This data suggests that, on average, the control participants initially made fewer errors during tracking tests compared to the CSM patients. Furthermore, the variance in the MediSens MAE scores was significantly lower in the control subjects (1.60) than the CSM patients (6.29). This large variability in the baseline MediSens MAE scores is consistent with reports by Manion et al., who demonstrated the presence of high variability in the baseline ODI scores between the control and the patients[25].

**Figure 5:**
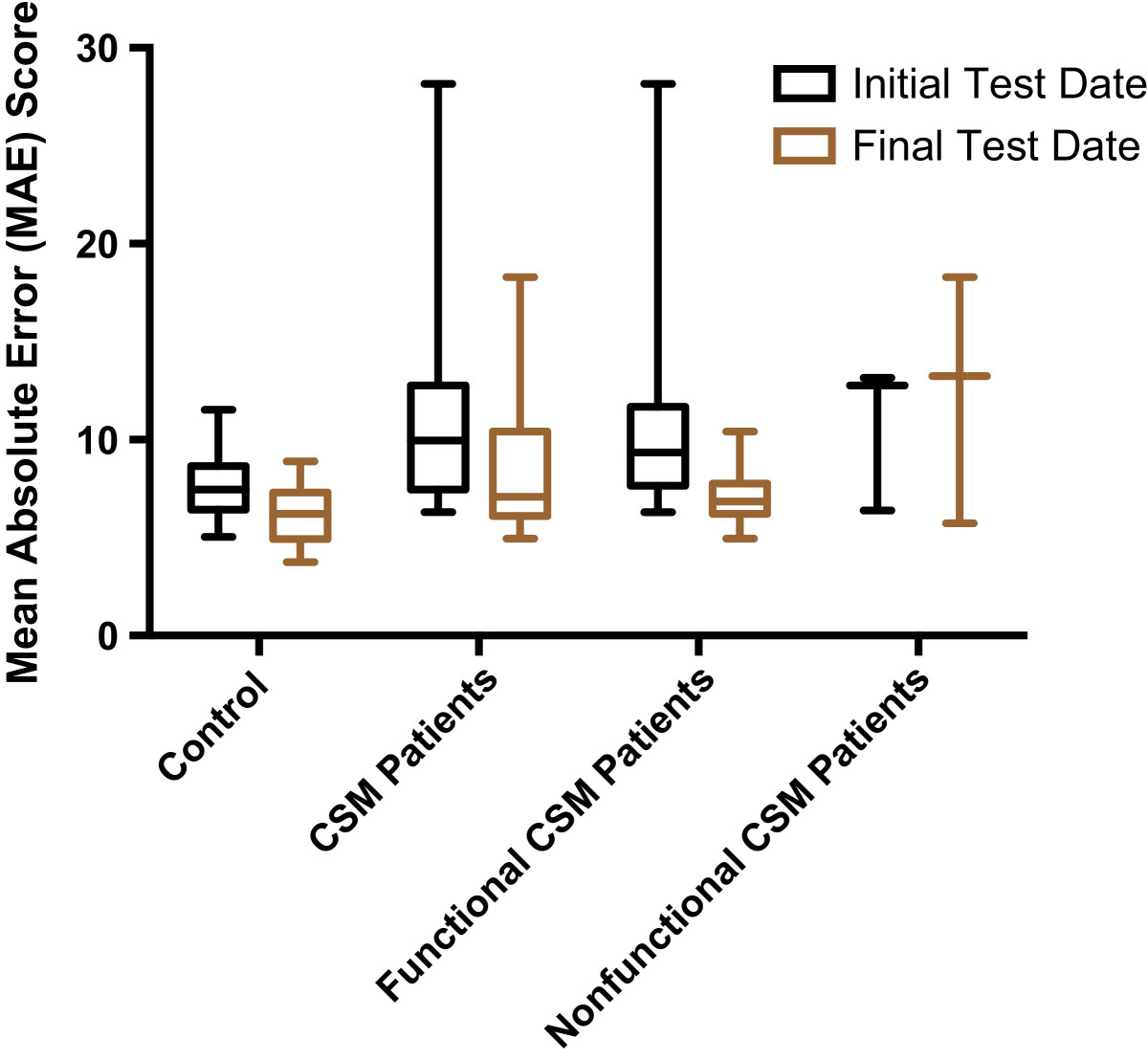
**The average MediSens MAE scores at the initial test date and the final test date were computed for control participants and CSM patients.** Based on final ODI assessment, CSM patients were separated into functional and nonfunctional groups, namely patients who improved after surgery and patients who did not improve after surgery. The preoperative and postoperative MediSens MAE scores were also compared for these functional and nonfunctional groups.

Table2 and Figure5 show that CSM patients’ average MediSens MAE scores improved to 8.71 ± 4.17 after surgery. The patients were further categorized into the functional and nonfunctional groups according to their postoperative ODI scores, as discussed in Section ‘ODI surveys’. The functional group demonstrated superior postoperative handgrip performance and less variability in the average MediSens MAE score (7.13 ± 1.60), compared to the nonfunctional group (12.41 ± 6.32). After further reviewing the past medical history of patients in the nonfunctional group, several factors were noted to be contributing to their lack of postoperative improvement. These factors included non-compliance with postoperative exercise restrictions and thrombophlebitis.

### Patient progress assessment with MediSens data

**Figure 6:**
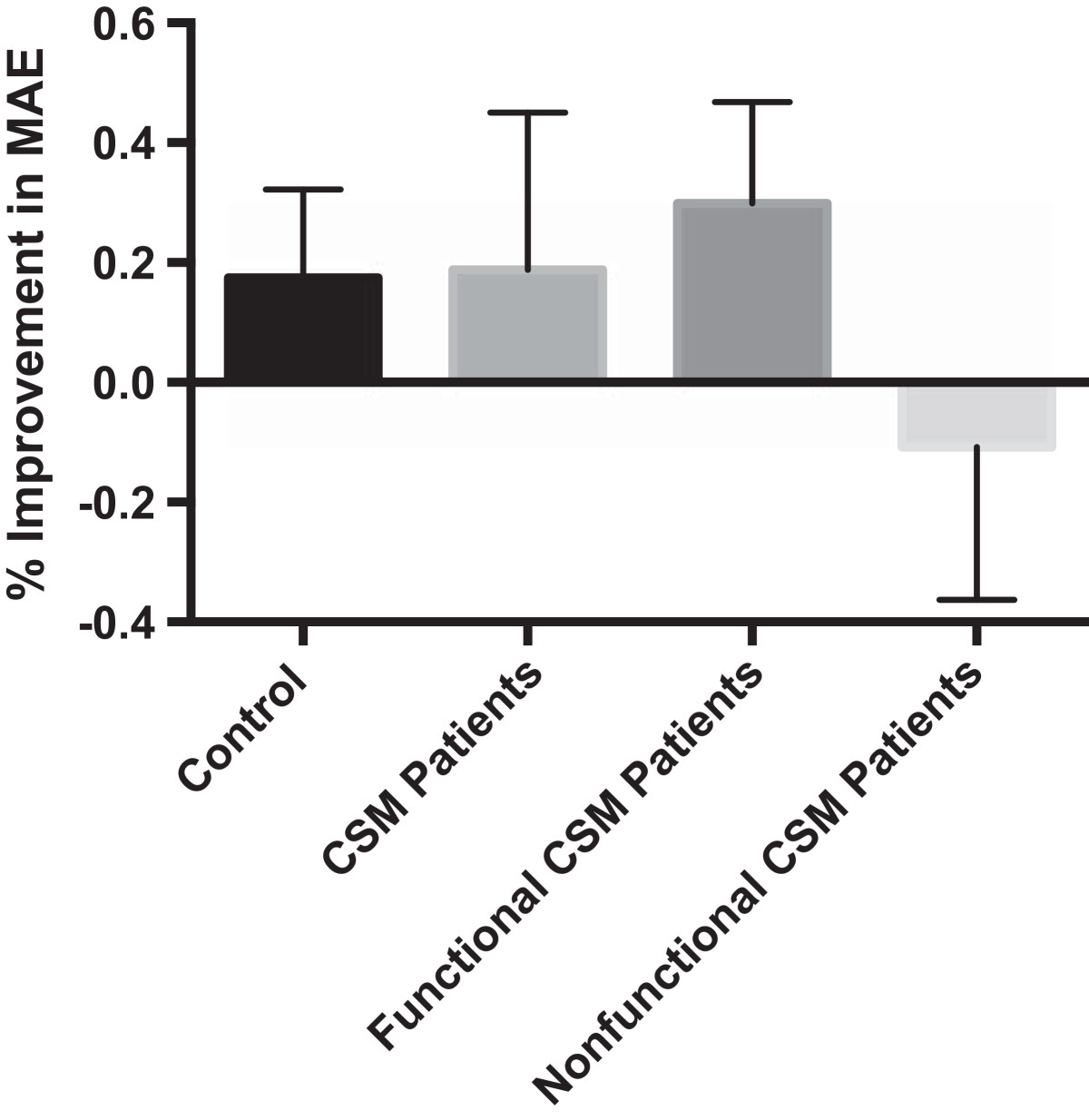
**Percent change in the MediSens MAE scores revealed motor improvement in both the control and pooled experimental groups.** Functional and nonfunctional groups are separated as previously described.

The MediSens handgrip tracking tests were performed preoperatively and the patients were followed for three months postoperatively. The patients were expected to improve their MediSens MAE scores during this time period secondary to motor learning. This is apparent in Table2 and Figure6, where both the control group and the surgical group improved in their average MediSens MAE scores throughout the course of the study.

Unfortunately, motor learning is an unavoidable confounding factor in tracking tests. We confirmed this learning effect in healthy subjects, which resulted in a slight improvement in the MediSens MAE score (*p*-value <0.0002) as summarized in Table2. The averaged MediSens MAE scores at the initial and the two-month attempts for the control group were 8.09 ± 1.60 and 6.21 ± 1.51 respectively. This data was used to account for the learning effect in the surgical group. The functional patients had a preoperative average MediSens MAE score of 11.46 ± 6.99 and a postoperative score of 7.13 ± 1.60, which showed a near-significant improvement (*p*-value <0.0640). The nonfunctional patients had a preoperative averaged MediSens MAE score of 11.24 ± 6.29 and a postoperative score of 12.41 ± 6.32, which showed no improvement (*p*-value <0.94). As illustrated in Figure6, these results demonstrate that both the control and the functional patient population had some degree of improvement in their hand neuromotor performance when compared to the nonfunctional patients.

In Table3, the percentage improvement of control participants was 17% ± 15%, whereas the functional group had an average percent improvement of 30% ± 18%. The 13% increase in improvement of the functional group, although not statistically significant (*p*-value <0.0834), suggests that the patients’ improvement was not due to the effects of learning alone. It suggests that their improvement in baseline motor function was the result of the surgical intervention.

In summary, the MediSens handgrip was able to differentiate between the expected motor learning and the postoperative improvement in handgrip neuromotor function of CSM patients. This suggests that it can be used to objectively quantify different levels of hand neuromotor function and to track postoperative rehabilitation.

### Correlation between subjective and objective data

Investigating the correlation of a new measurement to other existing measurements is a common method for validating the reliability and consistency of a new tool[13]. Here, we compared the improvement in handgrip performance (i.e., the MediSens MAE scores) with patients’ perceived levels of motor deficits in performing daily activities (the ODI scores). The Pearson product-moment correlation coefficient (or Pearson’s *r*) was used to quantify the level of correlation[26–28].

Table4 and Figure3(b) shows that the ODI and the MediSens MAE scores had a statistically significant correlation (*r*=-0.341, *p*<1.14×10^-5^). The Bland-Altman plot was used to assess the agreement between the MediSens MAE and ODI scores. The mean difference (i.e., the bias) was -0.26 (95*%* C.I. of -0.36 and -0.15) with an upper limit of agreement of 0.22 and a lower limit of agreement of -0.73. The plot showed a strong positive correlation between the mean and the difference of the two measures. This pattern is due to a variation in the ranges of the MediSens MAE and the ODI scores. That is, the MediSens MAE scores ranged from 0.049 to 0.28 while the ODI scores ranged from 0.21 to 0.94. This small range of the MediSens MAE and large range of the ODI scores accounts for the increasing differences as the averages get larger. However, the results are uniformly distributed within the limits of agreement, which indicates that there exists a considerable degree of agreement between the two scores.

Another means of comparing the MediSens MAE score with the ODI score is to correlate the percentage improvement of the two scores after receiving medical intervention. Table4 and Figure3(d) show that the percentage improvement in the MediSens MAE and the ODI scores had a significant correlation (*r*=-0.723, *p*<0.04).

Although the MediSens MAE score only quantifies hand neuromotor function, these findings demonstrate the potential use of tracking handgrip progress to help interpret and to complement surveys, like the ODI, that assess global neuromotor function.

### Subjective and objective tests for neuromotor assessment

We have demonstrated the ability of the MediSens handgrip to track patients’ postoperative hand rehabilitation. Tracking patient progress using patient-reported outcomes (e.g., the ODI) is difficult because of its subjective nature. Complications with the use of a subjective test to assess patient rehabilitation include misinterpretation of a survey question, adjusting responses to reflect a personal belief system, and offering biased responses to prevent “regret or self-blame” for undergoing surgery[25]. More recently, the phenomenon of “response shift” has offered further insight into the issues with tracking patient progress through patient-reported outcomes like the ODI[29]. Response shift refers to a changes in patients’ personal standards, priorities, and concepts toward healthy-related quality of life as a result of medical intervention[29]. Furthermore, Trachter’s study shows carpal tunnel syndrome that the patients’ reports of functional capacity were lower than the anticipated capacity[30, 31]. Fairbank et al. implicated “inconsistencies in answering questions [and] the natural fluctuations of symptoms as well as clinical improvements” as sources of errors[13]. Lastly, the use of changes in the averaged ODI scores for large-scale studies may overshadow inconsistencies in individual ODI results[25]. Such variability in ODI scores is implicit in the survey’s subjective nature and becomes problematic when assessing the patients’ motor progress[29]. Previous studies have demonstrated the usefulness of the ODI scores for assessing motor function, but more objective clinical tests are needed for tracking changes in motor function over time[13, 32, 33]. Objective testing instruments, such as the MediSens handgrip, may be a more accurate representation of an individual’s motor capacity because they eliminate the chances of subjective responses to questions.

Because this project was a pilot study, we encountered some difficulties. Our sample size created a difference in the male-to-female ratio between control and CSM patient groups, and it has underestimated the statistical power of the correlation between the MediSens MAE and the ODI scores. Despite the difference in the ratio of male to female, we have not found any statistically significant difference in the MediSens MAE scores within the demographic of control participants; there was no statistically significant difference between male and female participants (*p*-value <0.129), between weights at < 160 pounds and at ≥ 160 pounds (*p*-value <0.318), nor among all different permutations of age groups: 40s, 50s, 60s, and 70+. This reflects the efficacy of calibrating handgrip strength and that these demographics markers have not confounded the MediSens handgrip results. We acknowledge the limitation of a small sample size. However, this pilot study introduces the potential for a novel, objective, and quantitative test for assessing clinical progress of CSM patients. We hope this study will encourage research for elucidating other objective methods for assessing global neuromotor function.

## Conclusions

In this paper, we introduce the MediSens system as a novel instrument to identify neuromotor deficits. Our data suggests that the MediSens handgrip device can differentiate between normal motor learning and the postoperative neuromotor improvement in CSM patients. The MediSens handgrip scores may be used to supplement or replace the ODI surveys in CSM patients with hand neuromotor deficits.

## Authors’ information

Daniel C. Lu is an Associate Professor of Neurosurgery and Orthopedic Surgery at the University of California, Los Angeles. As the Director of the Neuroplasticity and Repair Laboratory and the UCLA Neuromotor Recovery and Rehabilitation Center, his research efforts are focused on modulating the neuronal circuitry and networks in the treatment of central nervous system disorders. Dr. Lu’s clinical expertise involves minimally invasive techniques in the surgical management of degenerative, traumatic, neoplastic, and spinal disorders. Majid Sarrafzadeh is a Distinguished Professor in the Computer Science Department and the Electrical Engineering Department at the University of California, Los Angeles. As the co-founder and co-director of the UCLA Wireless Health Institute (WHI), his research interests include embedded computing with emphasis on healthcare. Over the past 25 years, he has collaborated with many industries, recently co-founding MediSens and Bruin Biometrics.

## Authors’ original submitted files for images

Below are the links to the authors’ original submitted files for images. Authors’ original file for figure 1 Authors’ original file for figure 2 Authors’ original file for figure 3 Authors’ original file for figure 4 Authors’ original file for figure 5 Authors’ original file for figure 6

## Abbreviations

CSM: Cervical spondylotic myelopathy
ODI: Oswestry Disability Index
PD: Parkinson’s disease
MVC: Maximum voluntary contraction
MAE: Mean absolute error
RMSE: Root mean squared error.
