# Supplementary figures and images for: Utilization of a novel digital measurement tool for quantitative assessment of upper extremity motor dexterity: a controlled pilot study
Source: J Neuroeng Rehabil. 2014 Aug 13;11:121. doi: 10.1186/1743-0003-11-121 (PMC4138400; doi:10.1186/1743-0003-11-121)

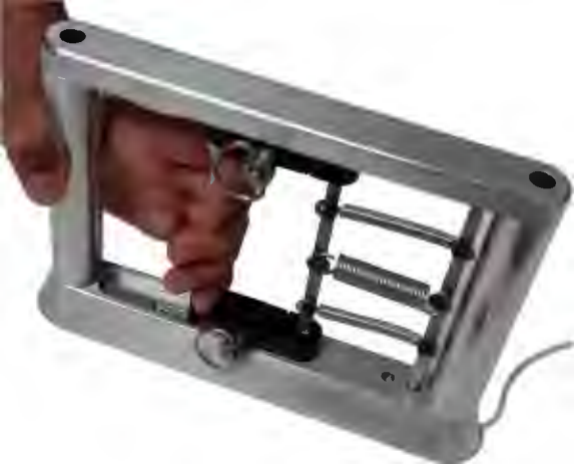

Supplement: Supplementary file 1 — Authors’ original file for figure 1 [file 12984_2013_642_MOESM1_ESM.pdf]

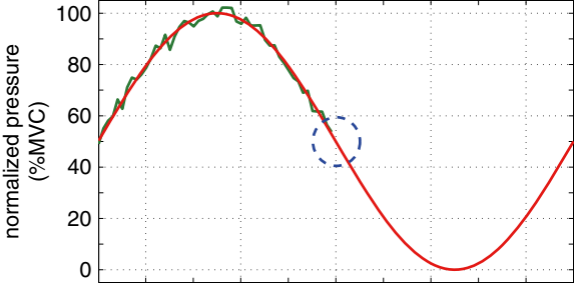

Supplement: Supplementary file 2 — Authors’ original file for figure 2 [file 12984_2013_642_MOESM2_ESM.pdf]

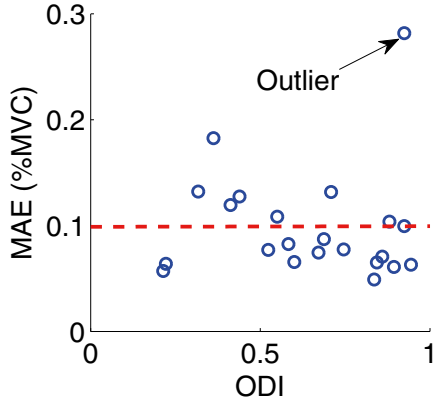

(a)

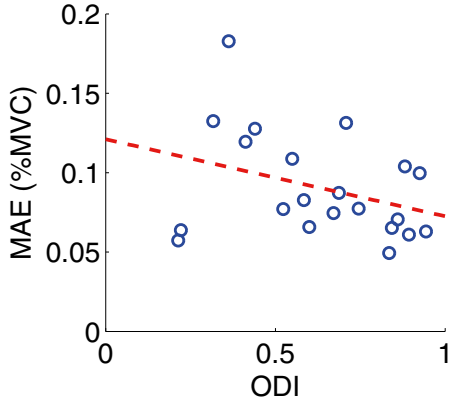

(b)

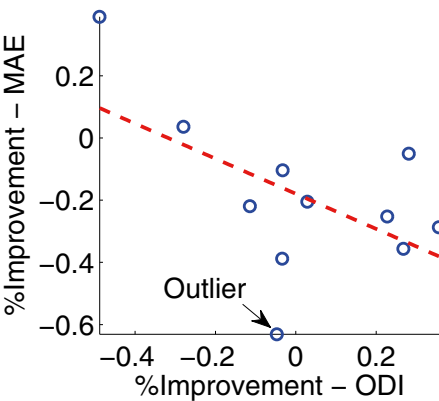

(c)

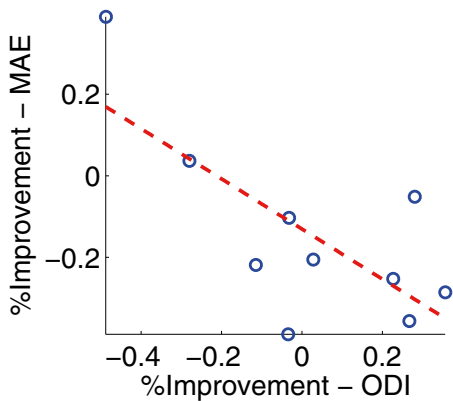

(d)

Supplement: Supplementary file 3 — Authors’ original file for figure 3 [file 12984_2013_642_MOESM3_ESM.pdf]

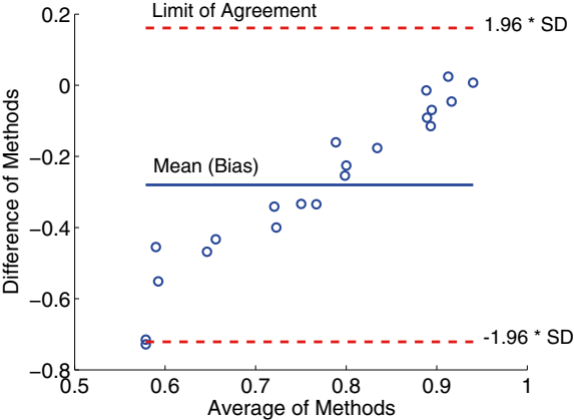

Supplement: Supplementary file 4 — Authors’ original file for figure 4 [file 12984_2013_642_MOESM4_ESM.pdf]

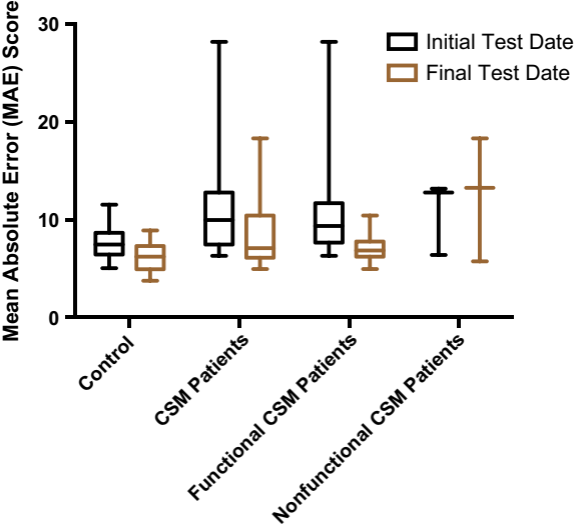

Supplement: Supplementary file 5 — Authors’ original file for figure 5 [file 12984_2013_642_MOESM5_ESM.pdf]

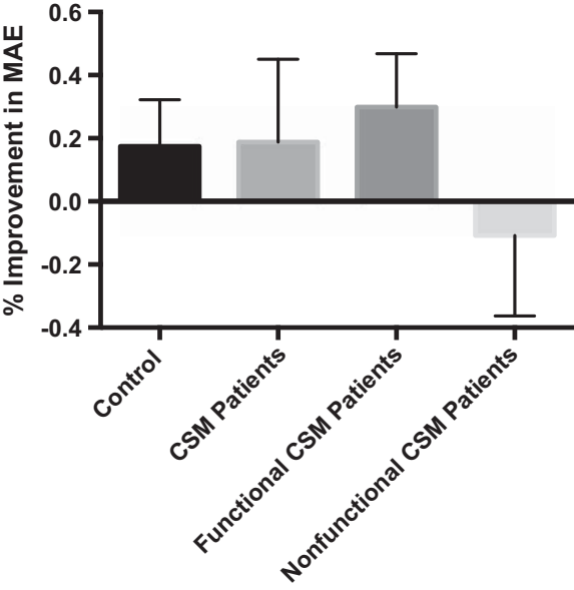

Supplement: Supplementary file 6 — Authors’ original file for figure 6 [file 12984_2013_642_MOESM6_ESM.pdf]
